# Annotated Dataset for Anomaly Detection in a Data Center with IoT Sensors

**DOI:** 10.3390/s20133745

**Published:** 2020-07-04

**Authors:** Laura Vigoya, Diego Fernandez, Victor Carneiro, Fidel Cacheda

**Affiliations:** Centre for Information and Communications Technology Research (CITIC), Campus de Elviña s/n, 15071 A Coruña, Spain; dfernandezi@udc.es (D.F.); victor.carneiro@udc.es (V.C.); fidel.cacheda@udc.es (F.C.)

**Keywords:** dataset, IoT, sensors

## Abstract

The relative simplicity of IoT networks extends service vulnerabilities and possibilities to different network failures exhibiting system weaknesses. Therefore, having a dataset with a sufficient number of samples, labeled and with a systematic analysis, is essential in order to understand how these networks behave and detect traffic anomalies. This work presents DAD: a complete and labeled IoT dataset containing a reproduction of certain real-world behaviors as seen from the network. To approximate the dataset to a real environment, the data were obtained from a physical data center, with temperature sensors based on NFC smart passive sensor technology. Having carried out different approaches, performing mathematical modeling using time series was finally chosen. The virtual infrastructure necessary for the creation of the dataset is formed by five virtual machines, a MQTT broker and four client nodes, each of them with four sensors of the refrigeration units connected to the internal IoT network. DAD presents a seven day network activity with three types of anomalies: duplication, interception and modification on the MQTT message, spread over 5 days. Finally, a feature description is performed, so it can be used for the application of the various techniques of prediction or automatic classification.

## 1. Introduction

The rapid eruption of new interconnected environments—smart homes and cities, cyber-physical systems, health systems, etc.—has caused the emergence of the Internet of Things (IoT).

The new environments substantially increase the number of devices that need light processors and simple network cards. At the same time, they implement different solutions to improve system stability, cost efficiency, fault tolerance and flexibility [1]. However, the Internet and its users are already under continuous attack, which represents a threat to the IoT as it incorporates many restricted devices. This fact can generate different points of view that generate new and ingenious malicious models. The challenge is to prevent the growth of such models or at least to mitigate and limit their impact [2].

In order to detect traffic anomalies, it is necessary to obtain a basic understanding of the performance and behavior of IP networks. This is the reason why information needs to be collected and processed [3].

On the one hand, supervised and unsupervised learning techniques have been incorporated into the treatment of performance, safety and scalability problems in these types of environments. Decision trees, random forest, neural networks and support vector machines are examples of these kinds of techniques. On the other hand, big data analysis deals with the variety, volume and need for processing that requires the automatic detection of problems almost in real time.

Therefore, having a dataset with a sufficient number of samples, contextualized, analyzed and with content annotations, is essential to understand the communications in this type of networks and to apply the techniques mentioned above.

The aim of this work is to present DAD: a complete and labeled IoT dataset for real-world traffic anomaly detection, with sufficient trace size, diverse anomaly scenarios and concrete feature extraction, which can be used for the detection of traffic anomalies in IoT sensor networks by using machine learning (https://github.com/dad-repository/dad).

The need for fast and efficient communications systems projects IoT as a new paradigm to offer important services. In fact, IoT introduces the need for specialized standards and communication protocols to handle typical resulting challenges. At the transport layer, TCP and UDP are the dominant protocols for most applications. However, several message distribution functions are required, depending on IoT application requirements. It is desirable that these functions are implemented in interoperable, standard ways.

Message Queuing Telemetry Transport (MQTT) protocol is the most widely used in IoT due to its low overhead and power consumption. It provides the connectivity between applications and users at one end, and network and communications at the other end. It is a publish–subscribe, extremely simple and lightweight messaging protocol, designed for constrained devices and low-bandwidth, high-latency or unreliable networks [4,5]. MQTT is the IoT protocol used in the scenario presented here.

### 1.1. Motivation

IoT environment is vulnerable to different security and privacy issues and various anomalies and attacks, which may cause unauthorized tasks to be performed by the remote malicious users [6]. With the development of IoT technology, information insecurity will directly threaten the entire IoT system. Thus, hackers, malicious software and viruses in the communication process might disturb data and information integrity [7]. In other cases, anomaly traffic on an IoT network may come from a wrong configuration, a abnormal installation or a hardware malfunction of the sensors.

The design of this scenario has been motivated by an incident that happened at the Centre for Information and Communications Technology Research (CITIC) [8]. An initial software failure, derived from incorrect sensor configuration in the data center, caused temperature sensors to send incorrect data to the cooling system. The temperature of the devices was affected, causing a hardware failure, and the entire system collapsed.

Creating a dataset from wireless sensor networks that reflects a real system, with light communications, using alterations in IoT protocols, will allow us to improve the detection of traffic anomalies in these environments.

### 1.2. Contributions of the work

The main contributions of this research are:

- How to model an IoT environment for the energy and environmental management of a data processing center.
- Building an annotated IoT dataset that facilitates the test with supervised learning algorithms.
- Performing an IoT dataset analysis to understand the normal and abnormal traffic behavior of the network.

### 1.3. Paper Organization

This work is structured as follows. The review of the literature is discussed in Section 2.

In Section 3, the scenario that was used to perform the data capture and create the dataset is presented, as are the physical structure of the real CITIC environment that motivated the project, both parametric and non-parametric mathematical models of the real data taken from the sensors and the design of the virtual environment adjusted.

Section 4 describes the dataset generation process. Once the system was implemented, controlled modifications in the scenario were accomplished on five of the seven days of the week. This was done with the aim of presenting different behaviors on the network, not only in certain types of anomaly, interception, modification or duplication, but also in the case of mixing these anomalies in the same period.

Dataset analysis and feature extraction are shown in Section 5. Finally, in Section 6, the results obtained are presented and discussed, followed by the conclusion of this document.

## 2. Related Work

This section is divided into three parts: a review of IoT datasets networks, and then an exploration of the different types of analysis and extraction of characteristics carried out in most popular datasets, to finally present the type of mathematical modeling and the statistical analysis that is usually carried out in sensor networks.

### 2.1. IoT Datasets

Although IoT is an emerging technology that has grown rapidly in recent years, the number of public datasets available for detecting traffic anomalies is limited. The datasets created from IoT networks will be presented hereafter.

In [9], the authors present Security Evaluation of Home-Based IoT Deployments (SoK), which is an IoT network with 45 devices used for a period of ten days, involving analyzing security properties for home IoT devices, and applying attack techniques and mitigation and stakeholders performing attacks on the IP protocol. The communication of the components has three attack categories: protocols, encryption and man in the middle (MITM). Twenty devices had one or more of their communication edges be susceptible to a MITM attack. The authors developed a dataset and their analysis focuses on a security evaluation—applying scoring rubrics to outline the weight distribution per property for each component.

The Cyber Range Lab of the Australian Centre for Cyber Security (ACCS) [10] designed a realistic Bot-IoT dataset and gave a detailed description of designing the testbed configuration and simulated IoT sensors. The environment incorporates a combination of normal and botnet traffic. The dataset includes probing attacks, denial of service and information theft. The subscribing and publishing IoT services is done via MQTT protocol.

Kitsuse is a plug and play network detection system with the capacity to detect attacks in different distributions [11]. In this case one of them belongs to an IoT network, wherein they establish a set of nine different devices connected to a Wi-Fi network and one of the cameras is infected with a real sample of the botnet Mirai malware.

Hyunjae et al. [12] created various types of network attacks in an IoT environment. Two smart home devices, and other devices, including some laptops or smart phones, were connected to the same wireless network. The dataset consists of 42 raw network packet files at different time points, containing different types of attacks: MITM (arp spoofing), DoS (SYN flooding), scan (host and port scan), UDP/ACK/HTTP flooding and Mirai. The dataset includes a good amount of network traffic, and one of its advantages is the number of attacks it presents.

A good dataset, necessary for validating and evaluating intrusion detection systems (IDSs), according to Bhuyan et al. [13], should monitor the daily situation in a realistic way; the labeling of traffic as benign or malicious must be perfectly labeled at both packet and flow levels for each piece of traffic, while assuring the labeling of each traffic instance must be correct.

Defining the optimal set of concrete features plays an important role for detection mechanisms. Besides, contemplating diverse sets of multistage attacks for dataset generation, allows one to go towards recent trends in security threats, and the ratio between normal and attack traffic should be taken into account. Among the aforementioned datasets, the first two [9,10] do not comply with completeness and correctness in labeling. The Kitsune [11] does not have diverse attack scenarios in the IoT network, while the last one mentioned [12] does not exhibit realistic behavior and concrete feature extraction. For these reasons, the construction and generation of a labeled dataset for Internet of Things that achieves the needed requirements for our purposes, became necessary.

### 2.2. Dataset Feature Analysis

When a dataset is created, it is important to perform an analysis and a feature extraction. The objective is to present the necessary information so that the communications can be understandable, and the dataset can be used for the application of prediction techniques or automatic classification.

In the literature we found a significant number of datasets for anomaly detection systems, with varied types of traffic and different attack scenarios, either built under a simulated or real environment, or by combining other different datasets. This subsection aims to show the works in which the selections of characteristics and feature extraction have been made. We selected the most used datasets in the literature and some examples of works that use this analysis to apply various algorithms.

In the case of the datasets presented for IoT, most of them also present feature analysis. The small number of these demonstrates the need for public datasets available for different IoT environments [9,10,11].

Three of the most widely disseminated datasets for the evaluation of networks based on intrusion detection systems and their description analysis are the KDD Cup 1999 Data [14,15], the NSL-KDD dataset [16] and the Darpa 2000 [17], which brings improvements over their previous 1998 and 1999 versions. The KDD Cup 1999 Data contains a large number of simple connections with 41 features and 24 types of attacks, and it was simulated under a typical LAN network used by US air forces. They performed nine weeks of acquiring TCP data by applying DoS, R2L, U2R and probing attacks. The NSL-KDD dataset aims to solve some inherent problems found in the KDD Cup 1999. This version still suffers from some problems discussed with the KDD and may not be a perfect representation of real networks due to the lack of public datasets for IDS networks. However, it has a large number of records, allowing for various experiments and using a wide range of algorithms [18,19].

The Darpa 2000 dataset simulates two LLdos attack scenarios (Lincoln Laboratory DDoS 1.0 and 2.0), and generates variations on the networks to present different attack scenarios. On the other hand, McHugh [20] then raises a series of shortcomings in the named datasets, where he emphasizes the lack of statistical evidence of similarity with typical network traffic, low traffic rates, relatively uniform distribution of the four main categories of attack and skewed distribution of the attacked hosts, among other things.

The project “Malware Capture Facility Project” [21], is a project whose main objective was to generate and capture anomalous activities (botnets) with specific characteristics. The project was responsible for analyzing, monitoring and capturing network traffic during some months of operation on different types of traffic and includes RRD files with the history of the traffic shape, two-way Argus flows (both the binary file and the file of text), web logs for all web traffic and a DNS report, among other things. The tags are generated manually by a group of security experts and are added to both the Argus files and the weblogs.

Bhuyan et al. [13] generated three different TUIDS (Tezpur university intrusion detection system) datasets: one for intrusion, one for coordinated scanning and a TUIDS DDoS both at the packet and stream level, with service traffic, web, email, samba, telnet, ftp and database, normal traffic of different users and 14 types of attacks distributed in six scenarios.

In real environments, Unibs [22] presents a dataset to classify encrypted traffic using SSH. It contains three consecutive days of traffic in business days, with 20 workstations in a local network. Likewise, the ISCX 2012 IDS dataset [23] has been generated by physically implementing a testbed using real devices and real traffic (SSH, HTTP, SMTP, IMAP, POP3 and FTP) through profiles which mimic the behavior of users in four different scenarios of malicious traffic. The University of Tokyo makes a dataset of traffic collected from KDD99, honeypots [24], which takes 24 significant characteristics into account and analyzes them.

Moustafa et al., built the UNSW-NB15 dataset [25]. This is not a IoT specific dataset, but it is applied to IoT environments [26]. The UNSW-NB15 dataset covers a collection of a large number of normal network traffic and malicious traffic instances. It encompasses realistic normal traffic behavior and combines it with nine attack categories: DoS, fuzzers, analysis, backdoor, Shellcode, worm, exploits, generic and reconnaissance [27] and its comparison with the KDD [28].

Other datasets were created or modified from the previously named ones [18,29,30,31]. These are intended to exploit the number of resources of the datasets already available, merging different types of attacks in the same dataset and this way taking advantage of the greater amount of information contained. The use of composed or modified datasets reduces the deficiencies found and improves feature extraction.

On the other hand, some works present the feature extraction for applications in machine learning [18,30,32]. The N-baIoT detects botnet attacks extracting behavior snapshots of the network, and using deep autoencoders [33].

### 2.3. Statistical Data Analysis

Mathematical modeling of sensors is one of the most efficient ways of knowing and predicting their behavior. To generate a dataset in an environment that is as realistic as possible, it is necessary to mathematically model the real environment using proven approach techniques. Many of the techniques used in this research have already been applied to similar environments.

Nikolova et al. [34] created libraries and proposed a methodology to adjust sensor data to specific models. They fit data to exponentials, Fourier series, Gaussian, polynomials, power series, rationals, sum of sines and Weibull distributions, using goodness-of-fit statistics, according to the shape and specificity of the sensor. The use of graphical and numerical measures, depending on sensor characteristics, number of data points and analysis requirements, allow them to perform the best. Analyzing other domains, the statistical methods used in the analysis of temperature data from composting have generally been based on essentially linear mathematics, Student’s t-test and analysis of variance with ANOVA. Shouhai et al. [35] realized the statistical modeling of a temperature time series from composting with a non linear mathematical model as the basis for statistical analysis. They realized a mathematical description of functions, such as the logistic, Gompertz, Richards and Weibull functions, to describe the temperature time series in composting. The goodness-of-fit of the model was evaluated using the R-squared (R2) statistic.

The assumption of Gaussianity is prevalent and fundamental to many statistical theories and engineering applications. In wireless sensor networks (WSN), Rassol et al. [36] have used different goodness-of-fit (GOF) tests to investigate the Gaussian characteristics of some specific data. The GOF test represents the measure of the compatibility of a random sample with a theoretical probability distribution function. They considered three graphical and five numerical GOF techniques to analyze the range estimation error data.

A similar approach to this work, related to the analysis of temperature data sensor, was done by Bhandari et al. [37]. They modeled the temperature phenomenon as a stochastic process and analyzed it using a time series modeling framework. The time series modeling approach selected was the autoregressive, integrated, moving average (ARIMA) model, which determines how the short-term predictability of future temperature is affected by sampling interval and extrapolation technique. The ARIMA models are used to forecast the remaining days of data. For each sampling rate, the ARIMA model is trained on three days of data, and then is capable of predicting up to two hours forward from that point.

## 3. Scenario

This section presents, in the first part, the structure of a network of sensors located in a real data center, the relationship between sensors, their distribution and their location. Likewise, the second part presents the mathematical modeling of the real data using validated statistical techniques and methods. The last part describes the implementation of the virtual scenario required for the construction of an IoT sensor network.

### 3.1. Physical Architecture

**Figure 1:**
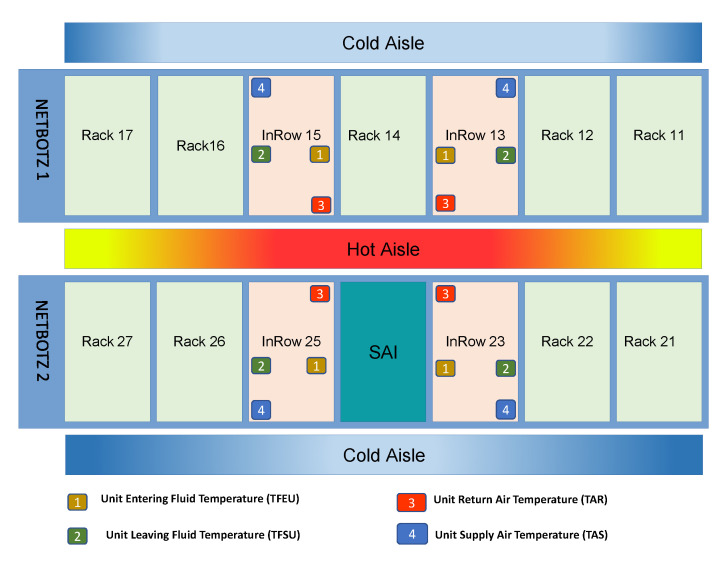
Physical architecture.

To approximate the dataset to a real environment, data were obtained from temperature sensors in the CITIC data center. These sensors monitor the temperatures of various elements of the datacenter through the use of intelligent passive sensors based on NFC technology. There are three elements with sensors: the racks, the power strips (PDU) and the refrigeration machines (InRow). We only considered the sensors of the InRows, because the values of the rest of the sensors are static, and therefore, are not significant. Since the positioning of the sensors is important to determining their function, the structure of the data center is shown in Figure 1.

NETBOTZ 1 with IP address 10.6.56.58 has five racks (11, 12, 14, 16 and 17) and NETBOTZ 2 with IP address 10.6.56.49 has four racks (21, 22, 26 and 27). Each rack has 2 sensors, one at the back, which is located on the side of the hot aisle, and one at the front, facing the cold aisle.

The most important elements on the system are the InRows (13, 15, 23 and 25), which are the devices responsible for cooling the air of the data center through a liquid cooling system.

The InRows have four relevant sensors:

- Unit supply air temperature **(TAS)**: This senses the temperature of the air coming from the cold aisle. Normally, the temperature measured by these sensors is related to the measurement obtained by the front sensors of the racks.
- Unit return air temperature **(TAR)**: This senses the temperature of the air located facing the hot aisle. The measure given by the sensor is equivalent to that measured by the rear sensors of the racks.
- Unit entering fluid temperature **(TFEU)**: This sensor measures the temperature of the system fluid before the cooling process.
- Unit leaving fluid temperature **(TFSU)**: This sensor measures the temperature of the liquid after the cooling process.

These sensors send data every five minutes, that is, 288 samples per hour, for a total of 4032 samples per sensor every day.

### 3.2. Mathematical Modeling Of the Sensors

**Figure 2:**
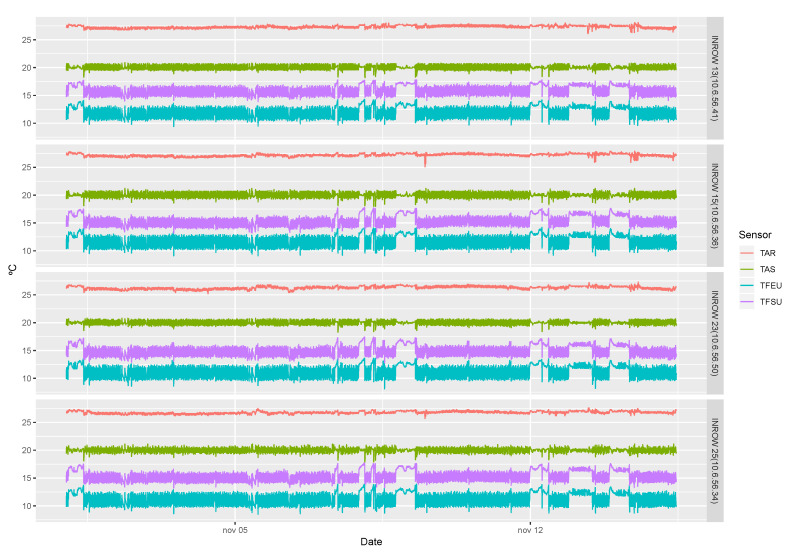
Signals of the sensors in the InRows.

For the analysis, a sample of 14 days sensor activity was captured. The signals obtained from the sensors in the data center are shown in Figure 2. The behavior of the signals determines the workload presented in the processors, since the temperature sensed depends on the machine’s workload. Some external events, such as opening a hall door, may also change the value of the sensors.

The purpose of this analysis is to characterize numerical data in order to understand the conceptual model of the observed values. To perform this task, firstly, we performed a descriptive statistical analysis of the data from the sensors.

Table 1 presents a summary of the statistics for the data by sensor and by InRow. This table shows how the average is around 20 ${}^{\circ }$C for the supply air sensor, 27 ${}^{\circ }$C for the return air, 11 ${}^{\circ }$C for entering fluid and 15 ${}^{\circ }$C for leaving fluid. The highest standard deviation is given by the fluid sensor that enters with a value of around 1 ${}^{\circ }$C. The lowest standard deviation is presented by the return air sensor.

**Figure 3:**
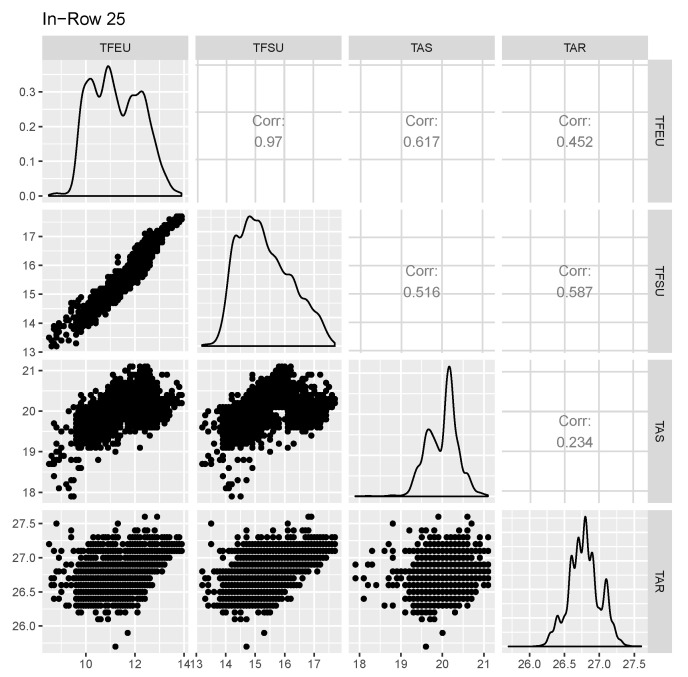
Sensor distribution and correlation: InRow 25.

Secondly, an analysis of the correlation between the sensors in the InRow has been made. Figure 3 displays the InRow 25 outcome, the Pearson correlation coefficient, in the upper triangle, distributions on the diagonal and a scattered plot in the lower triangle. The correlation between the fluid sensors is always high, as expected. There is also a small correlation between the supply temperature and the leaving fluid temperature. However, the return air temperature is poorly correlated with the other sensors, presenting a certain independence. The results for the other InRows are almost identical.

Parametric or non-parametric statistical methods can be used as mathematical models to estimate the quantitative behavior of the system. For this approach, we looked for a model that allows one to fix all the data over the same model.

A priori, taking into account Figure 3, we could assume that temperature signals follow a normal distribution. However, we have applied several methods to determine which distribution better fits the data. For this, we have performed different goodness-of-fit tests. To illustrate the process, the results obtained with InRow 25 are presented.

#### 3.2.1. Goodness-Of-Fit

The goodness-of-fit is a statistical hypothesis test aiming to determine how well a set of observed values fit a distribution. There are multiple methods for determining goodness-of-fit. Some of the most popular methods used in statistics are used in this section. A first approach was made by using normal, Weibull, lognormal and gamma distributions. For all the sensors, Kolmogorov–Smirnov and Anderson–Darling statistics are also computed, as shown in Table 2.

However, due to the p-values obtained, the decision is to reject the null hypothesis at a significance level of 0.05. In other words, the data do not fit the distributions considered. This is the reason why we have tried to approximate the series of data using a time series.

#### 3.2.2. Time Series Approximation

Anything that is examined sequentially over regular intervals of time is a time series. The aim is to estimate how the sequence of observations will continue into the future. The idea is to find a method to make a good fit to the selected data. We will start by applying simple linear regressions; then, an exponential smoothing and filter function; and subsequently, ARIMA methods to finally make use of seasonal and trend decomposition using Loess (STL) models.

#### Simple Linear Regression

In the simplest case, the regression model allows for a linear relationship between the forecast variable *y* and a single predictor variable *x*. It was intended to adjust data to a lineal, a quadratic term, a cubic term and an exponential term. The results obtained are shown in Table 3. For TAS sensor, null hypothesis cannot be rejected, so the sensor cannot be adjusted to a lineal regression. For other sensors the null hypothesis can be rejected. However, the values given by the R2-Adjusted are too low and MSE too high in the majority of cases. That means that the data cannot be fixed to the model.

#### ARIMA Model

ARIMA models provide another approach to time series forecasting. It is one of the most widely used approaches to time series forecasting, and provides complementary approaches to the problem. ARIMA models aim to describe the autocorrelations in the data. Along with looking at the time plot of the data, the ACF plot is also useful for identifying non-stationary time series.

**Figure 4:**
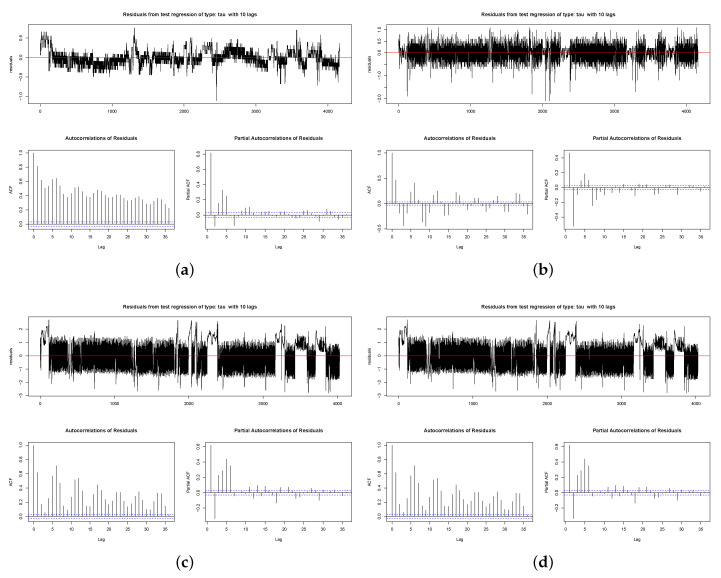
Signal, autocorrelations and partial autocorrelations of residuals: (**a**) TAR sensor. (**b**) TAS sensor. (**c**) TFEU sensor. (**d**) TFSU sensor.

The autocorrelation plot, shown in Figure 4, lets us know how the given time series is correlated with itself. For a stationary time series, the ACF will drop to zero relatively quickly, as the TAS sensor does.

The partial autocorrelation at lag *k* is the correlation that results after removing the effect of any correlations due to the terms at shorter lags. Differencing is a method of transforming a non-stationary time series into a stationary one. This is an important step in preparing data to be used in an ARIMA model.

As observed in Figure 4b, the autocorrelation of residual for TAS sensor shows that it is a stationary one, so the ARMA model is used. That is to say, for the other sensors it is necessary to differentiate the series, using ARIMA.

The auto arima tool in R allows us to improve the precision and agility of the ARIMA calculation. The results obtained are shown in Table 4.

Despite making use of the principle of parsimony, the high number of parameters obtained with auto arima will require a very high computational cost that can cause problems in the implementation.

#### STL Decomposition

Time series data can exhibit a variety of patterns, and it is often helpful to split a time series into several components, each representing an underlying pattern category. Each data point (*Y*${}_{t}$) at time *t* in a time series can be expressed as either a sum or a product of three components; namely, seasonality (*S*${}_{t}$), trend (*T*${}_{t}$) and error (et) (also known as white noise). For additive time series,

(1) $$Y_{t}=S_{t}+T_{t}+\epsilon_{t}$$

STL is a versatile and robust method for decomposing time series. STL is an acronym for “seasonal and trend decomposition using Loess”; Loess is a method for estimating nonlinear relationships. The STL method was developed by Cleveland et al. [38].

STL has several advantages over other classical decomposition methods [39] and handles any type of seasonality. The seasonal component is allowed to change over time; the rate of change and the smoothness of the trend-cycle can be controlled by the user. It can be robust to outliers (i.e., the user can specify a robust decomposition), so that occasional unusual observations will not affect the estimations of the trend-cycle and seasonal components. However, they will affect the remaining component.

**Figure 5:**
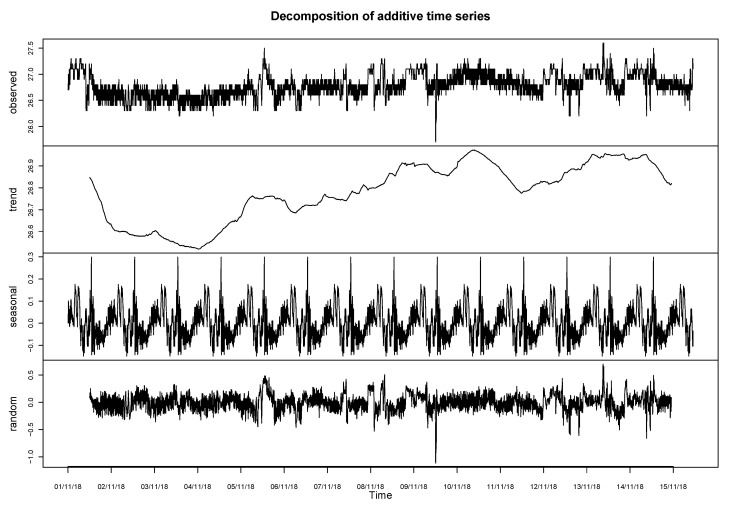
STL decomposition for TAS sensor.

Therefore, the method selected to generate the temperature data is a STL decomposition method. The selected sampling frequency is 288 samples per day. As shown in Figure 5, three vectors of data are extracted, one that corresponds to the trend, one to the seasonality and one of random values, and these are added by the algorithm as presented in Equation (1).

### 3.3. Virtual Scenario Description

**Figure 6:**
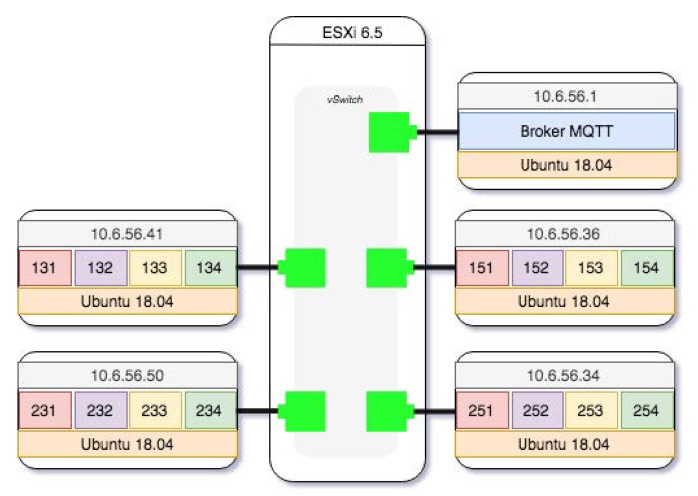
Virtual scenario.

The virtual infrastructure necessary for the creation of the dataset was formed by a VMWare ESXi6.5 virtualization environment on which a virtual network, called internal IoT, has been defined. This virtual network is isolated from the rest of the infrastructure networks, using the capacity of VSwitch that this tool provides. About the virtualized environment, five virtual machines with Ubuntu server 18.04 operating system have been created, each of them connected to the internal IoT network, see Figure 6, so that the traffic between them is totally isolated. On one of these machines, the mosquitto [40] broker has been installed, which centralizes the subscription of all MQTT clients and is the place where the traffic capture is carried out with tcpdump [41]. In this last machine, the process of deployment and provisioning of the code that implements the generation of MQTT communications from the clients takes place. This process initializes the clients; distributes the code associated with each node that implements the assigned time series; and initializes each node and controls its execution until the sending of packets to the broker ends. In each of the four client nodes, a process is launched that simulates each of the four sensors that make up each of the four refrigeration units. These processes receive an identifier correlated with the cold unit identifier (InRow); for example, the cold unit 13 has processes 131, 132, 133 and 134, each simulating the operation of the unit supply air temperature (TAS), unit return air temperature (TAR), unit entering fluid temperature (TFEU) and unit leaving fluid temperature (TFSU). Each of the samples generated by the sensors is sent every five minutes to the broker by means of a MQTT message that contains its node identifier as ClientId, once the sensor has been connected for publication of the corresponding topic.

In the broker, through tcpdump, a capture of the traffic exchanged with the client nodes is made. It is later written down and noted as part of the information that the clients have about the sending of the tokens in the connection, allowing them to mark those tokens that belong to anomalous situations.

## 4. Dataset Generation

Although good datasets are necessary for validating and evaluating IDSs, generating such datasets is a time consuming task. According to Bhuyan et al. [13], we present the following process of generating DAD via monitoring seven days of the situation that occurs daily in a real environment. The labeling of traffic as benign or malicious is done at both packet and flow levels for each piece of traffic, with a sufficient trace size and with a concrete feature extraction presented also in this work. DAD presents diverse sets of anomaly scenarios where abnormal traffic is statistically different from normal traffic, when the majority of network traffic instances are normal.

### 4.1. Dataset Setup

As indicated in Section 3.3 within the virtual infrastructure, four client nodes have been configured with four processes, and each process simulates a sensor by sending temperature samples to the broker. Following the MQTT publisher–subscriber terminology, in each node there are four MQTT clients publishing their messages (tokens) to a centralized broker. Therefore, for each of these sensors a code has been implemented that allows the publication of MQTT messages. Algorithm 1 illustrates these processes.
**Algorithm 1 **Client operationInitialize client (clientId, topic) Set the number of tokens, the number of waiting seconds and the pattern identifier Create the MQTT client (brokerAddress, clientID, PERSISTENCE) Establish connection with the broker (communication
options) **for** number of tokens **do**    Calculate temperature to send according to pattern    Post MQTT message with topic    Wait for token transmission confirmation **end for** Disconnect MQTT client Destroy MQTT client


First, the client is configured to use a clientID and publish its messages to a certain token. Later, the client is created that indicates the broker’s IP address and the transfer mode (persistent, in this case). Then, it is prompted for communication options and it starts sending messages (tokens) based on the number of messages that are passed as an argument. To reflect the actual behavior of the infrastructure in which the temperature samples were taken, MQTT clients do not implement any of the authentication and transfer security mechanisms between the sensors and the broker.

In order to implement the traffic anomalies, the behavior of the node can be modified in one of the following ways:

- **Interception**: randomly deleting some sent packets.
- **Modification**: changing the temperature to be sent, without following the established pattern.
- **Duplication**: sending more tokens than the number initially planned.

For the calculation of the temperature to be sent, an algorithm that implements the time series described in Section 3.2 Equation (1) has been coded. For each token, a sum computes from the addition of three values: the seasonal pattern value of that node, the trend temperature pattern value and a random value (that simulates the operation of the real environment). When a modification behavior is implemented, the temperature standard value is modified randomly.

Subsequently, the confirmation of the transmission of the token and the configured time of spacing between tokens are expected. In the replication anomaly simulation, this time is reduced randomly, resulting in more tokens being sent than usual. In the case of the simulation of the interception anomaly, the waiting times for sending the token must be modified to avoid timeout problems.

DAD comprises some tokens with those modifications and others without any of them. It is labeled at packet level, indicating the type of token (normal or anomaly). A CSV document and an XML document are used in which the following characteristics are indicated for each packet:

- **frame.number**: frame number in the PCAP package.
- **ip.src**: MQTT client IP.
- **tcp.srcport**: MQTT client TCP source port.
- **mqtt.clientid**: MQTT client identifier.
- **mqtt.msgid**: Token identifier or message number.
- **label**: 0 indicates that it is a packet without anomalies and 1 that the packet is part of a flow that has been altered.

### 4.2. Capture and Structure Methodology

Through a process distributed and synchronized with a NTP server, a network of sensors is created where the publication of samples is collected in a central broker. The capture of the packets is carried out in the network interfaces of the node in which the broker is running. The simulation process has various tools that allow the following operations:

- Deploy and configure the sensors in the nodes: it allows the configuration of the number of nodes and sensors (clients) that will participate in the simulation.
- Verify the status of execution of the configuration of sensors in the nodes: it allows the verification of the state in which each of the sensors is, in the different nodes of the simulation.
- Make the sensor operation traceable: it is also possible to track the operation of the different sensors, recording the activity of each of them in a corresponding log file.
- Launch the execution of different processes: automation of the execution of the different processes involved in the simulation.
- Parameterize the number of tokens to send and the periodicity of the shipments.
- Parameterize the identifier to be sent as clientId.
- Generate the sample that corresponds to the pattern of the day of the week.
- Cancel or reset the simulation at any time during the operation.
- Program its operation asynchronously.

All these samples are captured by means of a sniffer process located at the broker interfaces.

### 4.3. Labeled Process

Finally, once the traffic has been captured at the broker, a dataset annotation process is performed to identify which packets are part of flows with an abnormal behavior. This information has been previously generated at the client nodes. The annotation process is performed using the Scapy [42] tool. A pseudocode describing this process is specified in Algorithm 2.
**Algorithm 2** LabelingCreate XML and CSV headers **for** each packet in PCAP file **do**    **if** haslayer(TCP) **then**        Get IP address and TCP source port    **end if**    **if** haslayer(MQTTPublish) **then**        Get msgid, clientId and topic        **if** clientId in anomaly sensor **then**           Annotate package as anomaly        **end if**        Build line with frameNumber, $ip_{s}rc$,$tcp_{s}rcport$,clientID, msgid, label    **end if**    Record information in CSV and XML format **end for**


Using the Scapy tool, the different frames are traversed, obtaining the necessary data to record the dataset, the IP address and TCP port of the MQTT client. If it is an MQTT packet, the message or token identifier is obtained, and the client identifier indicated in the packet. Finally, those packets that are part of streams that have been modified are marked in the label field.

Next, there is an example of annotation in the CSV file:

frame.number;ip.src;tcp.srcport;mqtt.clientid;mqtt.msgid;label

1;10.6.56.34;38378;131;1;1

## 5. Dataset Analysis

DAD is a labeled dataset consisting of seven days of network activity with anomalous packets spread over five days. It has three different types of anomalies: duplication, interception and modification on the MQTT message. For the analysis, some traffic features have been extracted [43].

The anomaly traffic is made from InRow 13 with IP source 10.6.56.41. The anomaly has been done in specific days of the week. The distribution is as follows:

- Monday 21st: there are no anomaly packets.
- Tuesday 22nd: some packets have been removed, so packets are not labeled as anomaly.
- Wednesday 23rd: a modification of packets is made between 4 and 6 h.
- Thursday 24th: insertion of packets in less than 5 min at 3 h.
- Friday 25th: a mix of interception, duplication and modification is done at 6 h and between the 14–16h.
- Saturday 26th: a mix of interception, duplication and modification is done at 6 h and between the 14–16h.
- Sunday 27th: there are no anomaly packets.

### 5.1. General Dataset Description

To know the behavior of the dataset in a generic way, some characteristics that are considered important in the detection of traffic anomalies, such as numbers of source and destination bytes, numbers of source and destination packets sent, the number of packets for each protocol and also the numbers of abnormal and normal packets by day are analyzed below.

DAD has a total of 101,583 packets. It presents UDPand TCP traffic on the transport layer and MQTT as the IoT protocol. The 96.9% of the traffic corresponds to TCP packets, and 3.4% to UDP traffic. The 63.3% of the total are MQTT packets and 16% of these packets are anomalies. The detailed information is presented in Table 5.

This table also shows that interception anomalies have not been marked. This is because the modified packets have been removed, so their percentage cannot be reflected in the general statistic about packets marked as abnormal. In other words, the anomalous traffic percentages do not take into account the interception anomalies.

**Figure 7:**
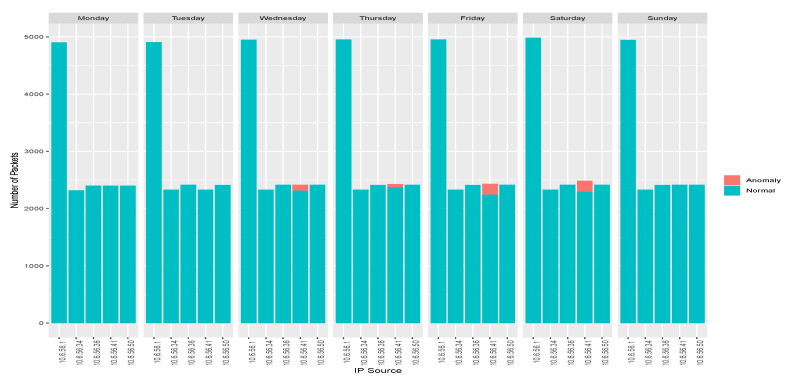
Number of packets by IP source by label and by day.

One of the features that provides a wealth of information in traffic analysis is the number of packets sent by each IP address source. This information allows one to gain an insight into the distribution of the packets over the network and predicts its behavior. The number of packets sent from every source IP over the days is shown in Figure 7. Every IP address represents an InRow, that is, the packets presented are the sum of the packets sent by the four sensors.

Due to its own structure, and to the publish–subscribe messaging transport qualities of the MQTT protocol, an IoT sensor network sends all packets to the broker, and the sensors do not create connections to each other. Besides, the largest amount of package shipping will be done by the broker. In fact, the connections of the sensors to the broker present the same distribution as expected.

The number of packets sent, and the relationship between packets and the number of bytes sent for normal traffic are homogeneous. The node with IP address 10.6.56.34 has a second network interface through which it synchronizes with the NTP server. For this reason, UDP traffic from this node does not appear, and this can be seen in the absence of some packets in the node. On the other hand, unbalanced packet distributions on IP address 10.6.56.41 represent abnormal network behavior.

Another relevant feature is the distribution of packets per protocol. IoT introduces the need for specialized standards and communication protocols to handle resulting challenges. At the transport layer, TCP and UDP are the dominant protocols for most of the applications, while MQTT is the most widely used in IoT due to its low overhead and power consumption [4]. The dataset presents UDP and TCP protocols at the transport layer. It also presents NBNS and NTP protocols over UDP and MQTT over TCP. Table 6 shows the number of packets on each protocol. The packets that are found in the TCP box with blank space are packets sent over TCP only. As mentioned above, all anomaly packets are over MQTT messages. Therefore, the other packets have normal behavior.

In addition to the analysis of packets, an analysis of flows is performed. Flows are established as unidirectional, taking an idle timeout of 30 s (typical timeout values range from 15 s to 5 min [44]). This way, a TCP connection is divided into different flows. Each of the sensors set up a TCP connection with the broker on a daily basis. The connection always closes the next day, so some packets corresponding to a flow of one day will be presented the next day. The labeling of the flows was performed a posteriori. All packets belonging to the same flow were marked as anomalies. The general statistics on the number of flows by day are presented in Table 7. The dataset has a total of 67,848 flows, of which 544 correspond to anomaly flows. The number of flows during the days is homogeneous, presenting a smaller number on Sunday due to connection closures that are made the next day. All anomalies are carried out on TCP flows.

### 5.2. TCP/MQTT Description

Next, a description of TCP packets is presented. They contain the MQTT and all abnormal packets. As can be seen in the general analysis, and due to IoT conditions, most of the characteristics present homogeneity in their behavior. This situation led us to look for IoT features that allow us to establish specific traits in the behavior of the network.

**Figure 8:**
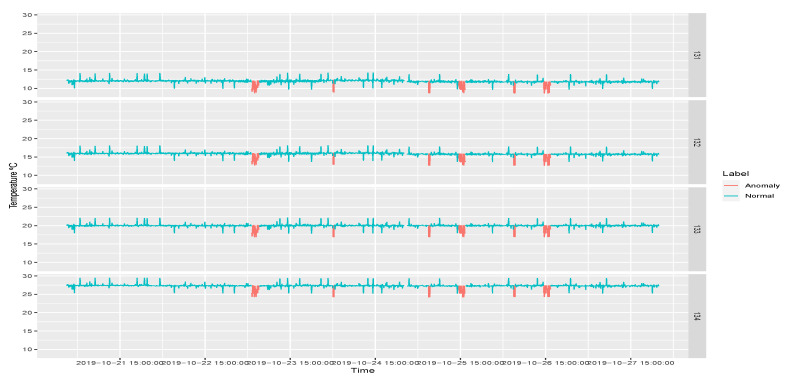
InRow 13 sensor signals.

The MQTT payloads are extracted and grouped according to the clientId to recreate a time series and to be able to visualize the temperature signal presented by the sensors in the dataset. Figure 8 shows the sensor signal pf both normal and abnormal traffic, over the seven days as a time function.

All InRow sensors have the same IP address, but the ports used for message transmission are different for each of the sensors. Therefore, a total of 16 ports are used per day that are assigned with each connection. This means that a total of 112 TCP ports are used.In each connection, the sensors receive a token with the clientId, through a certain port, which determines the sensor identifier. A clientId equivalence table is made allowing to know which port each sensors is using at the time of connection. Each sensor uses one port a day and changes with each connection. This is the reason why a total of 112 TCP ports are used.

**Figure 9:**
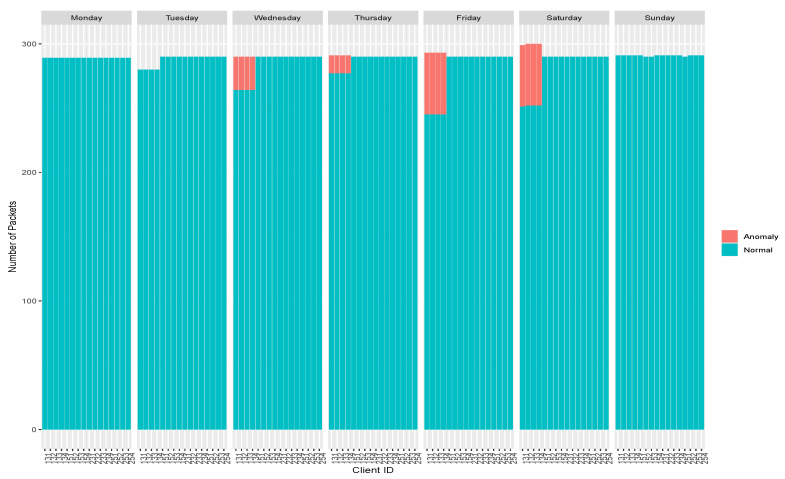
MQTT clientId.

As we know, the total traffic distribution packets are uniform, so we expect the same behavior from clientId packets under normal conditions. The number of packets sent by day by clientId is shown in Figure 9. As mentioned above, a first instance to detect unlabeled anomalies is the lack of uniformity in traffic. This means that interception anomaly are detected through the absence of packets on Tuesday. As mentioned before, some missing Sunday packages correspond to the connection closures that are presented the next day.

Initially, all flows should be identified and labeled as normal and abnormal. A unique TCP connection is made throughout the day, so flags are analyzed to understand the state of the connection. The analysis of the flags was carried out to determine how the broker established and closed the connections, as shown in Table 8. The behavior of these particular connections is due to the fact that at the application level, it sends the disconnection message but then does not wait for the ACK flag.

**Figure 10:**
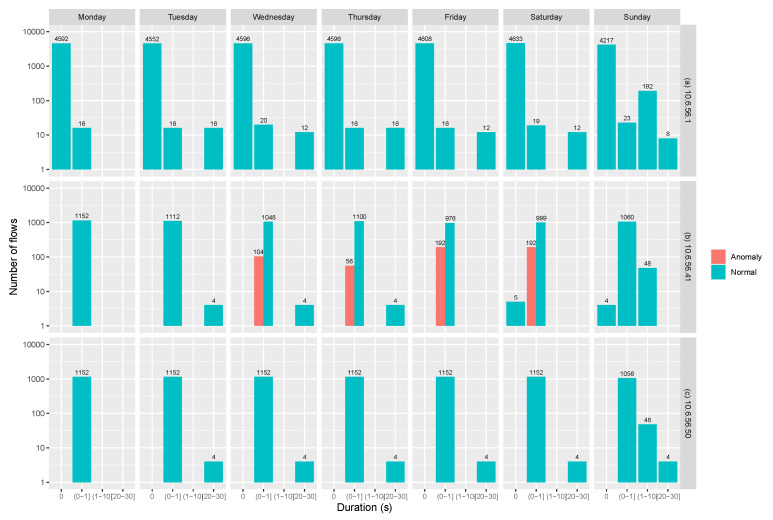
Log-linear flow duration. (**a**) Broker. (**b**) An abnormal node. (**c**) A normal node.

Another relevant feature to analyze is flow duration, described in Figure 10. This figure presents the behavior of the broker with IP address 10.6.51.1, an InRow with IP address 10.6.56.50 which exhibits normal behavior, and an abnormal InRow with IP address 10.6.51.41.

**Figure 11:**
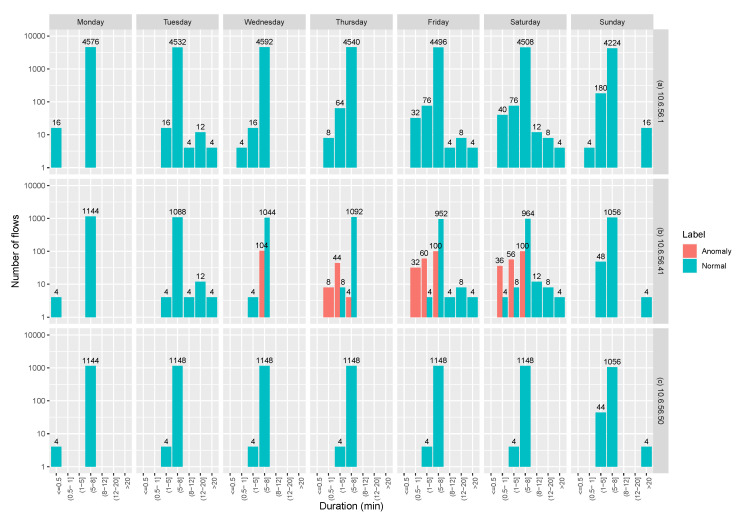
Log-linear duration of lag between flows. (**a**) Broker. (**b**) An abnormal node. (**c**) A normal node.

Interception anomalies are distinguished by avoiding the reception of some packets. Recognition of this anomaly is difficult, but one of the mechanisms for its identification is through the arrival time between flows of the same sensor. Figure 11 shows a log-lineal representation of the lag duration between flows. It presents the behavior of the broker with IP address 10.6.51.1, an InRow with IP address 10.6.56.50 which exhibits normal behavior and an abnormal InRow with IP address 10.6.51.41.

The time-lag established in the reception between one message and the other is five minutes. When an irruption anomaly is carried out, this time is altered, become greater than that determined by the system, and therefore, the number of packets decreases. The appearance of duplication anomalies, together with the interception anomalies, can make the presence of the last one difficult to detect. However, since duplication anomalies are marked in the dataset, the presence of interception anomalies must occur between normal flows.

The presence of flows with a duration greater than 5 min is evidence of flows corresponding to this type of abnormally, as is shown in Figure 11. According to this, the interception anomaly is done on Tuesday, Friday and Saturday as expected. This feature allows us to label all those flows that correspond to this kind of abnormal behavior.

DAD allows the evaluation of anomaly detection algorithms. A set of features was selected among the most used features in this kind of domain [30,32,45]. These features can be used for the application of different machine learning techniques.

## 6. Conclusions

The huge number of devices connected to the network, and the increase of connected elements in an IoT network, with generally limited capacity, offer a series of advantages with respect to resources, lighter protocols, greater exchange of messages and simpler topologies. However, this greater connectivity extends the vulnerability of the services and the possibilities to different network failures and in the same way simplifies the anomalies in network traffic. The initial step in preventing or avoiding network failures is to detect those anomalous behaviors in network traffic.

In this paper, we have presented DAD, a labeled IoT dataset for anomaly detection, with real world traffic, sufficient trace size, diverse anomalies scenarios and concrete feature extraction that facilitates testing with machine learning algorithms.

These algorithms will allow the detection of anomalies that arise from IoT devices. After a review of the literature, we did not find a dataset that suited our needs, which motivated us to generate this dataset, which aims to fill the gaps to our requirements (amount of traffic, correct labeling, IoT protocols represented, capture duration, etc.) and that may be useful to other research groups, too.

In this process, real data were taken from the temperature sensors of the CITIC data center, where we have realized that the modification of the data on IoT networks can substantially affect all the behavior of the refrigeration system of the data center. These real data were mathematically modeled by applying both parametric and non-parametric methods to approximate the real distribution.

After exploring the most used techniques for the mathematical model function, time series using STL decomposition fitted the real data better. This happened because it took distribution, past events, stationarity and seasonality in the presented data into account.

The modeled data have been implemented in a virtual environment, which emulates five devices, a broker and four InRows. The experimentation performed represents the real environment in a reliable way.

Most systems for detecting traffic anomalies show the alterations on network packets. In this case, because of the type of networks being managed, we modified to the IoT protocol payload, at the application layer. In this specific case, the use of the MQTT protocol was selected, given that it is the protocol most used in IoT networks.

A detailed analysis of the dataset has been reflected in this article. This fact makes DAD a versatile dataset, easy to follow and manipulate, to facilitate the work of other research groups that want to use it in their projects. It contains 67,848 flows and 101,583 packets. In total, 3.4% of these packets are UDP and 96.9% are TCP. It is also important to note that the unidirectional flows and the packets were marked. Finally, 0.8% of these flows were tagged as anomalous.

## Figures and Tables

**Table 1:** Statistical information.

Device	Sensor	Mean	SD	min	max	Median
INROW 13 (10.6.56.41)	TAS	20.00	0.38	18.20	20.90	19.90
	TAR	27.33	0.24	26.10	28.10	27.30
	TFEU	11.94	0.93	9.40	14.30	11.80
	TFSU	15.89	0.79	13.90	17.80	15.80
INROW 15 (10.6.56.36)	TAS	20.00	0.42	18.00	21.20	19.90
	TAR	27.20	0.25	25.10	27.90	27.20
	TFEU	11.69	0.98	9.10	14.20	11.60
	TFSU	15.37	0.89	13.20	17.80	15.20
INROW 23 (10.6.56.50)	TAS	20.00	0.34	18.30	20.80	20.00
	TAR	26.29	0.27	25.20	27.30	26.30
	TFEU	11.14	1.00	8.10	13.60	11.00
	TFSU	14.95	0.88	12.80	17.40	14.80
INROW 25 (10.6.56.34)	TAS	20.00	0.37	17.90	21.10	20.10
	TAR	26.79	0.22	25.70	27.60	26.80
	TFEU	11.24	1.00	8.50	13.90	11.10
	TFSU	15.34	0.88	13.20	17.70	15.20

**Table 2:** Goodness-of-fit statistics.

	Normal		Lnorm		Gamma		Weibull	
	**D/An**	***p*** **-Value**	**D/An**	***p*** **-Value**	**D/An**	***p*** **-Value**	**D/An**	***p*** **-Value**
Unit Supply Air Temperature **(TAS)**								
Kolmogorov-Smirnov statistic	0.15595	<$2.2\times 10^{-16}$	0.15884	<$2.2\times 10^{-16}$	0.1579	<$2.2\times 10^{-16}$	0.12464	<$2.2\times 10^{-16}$
Anderson-Darling statistic	47.257	$1.44\times 10^{-7}$	49.244	$1.44\times 10^{-7}$	48.562	$1.44\times 10^{-7}$	51.504	$1.44\times 10^{-7}$
Unit Return Air Temperature **(TAR)**								
Kolmogorov-Smirnov statistic	0.11654	<$2.2\times 10^{-16}$	0.1149	<$2.2\times 10^{-16}$	0.11544	<$2.2\times 10^{-16}$	0.16636	<$2.2\times 10^{-16}$
Anderson-Darling statistic	45.533	$1.44\times 10^{-7}$	45.228	$1.44\times 10^{-7}$	45.322	$1.44\times 10^{-7}$	94.882	$1.44\times 10^{-7}$
Unit Entering Fluid Temperature **(TFEU)**								
Kolmogorov-Smirnov statistic	0.082724	<$2.2\times 10^{-16}$	0.075811	<$2.2\times 10^{-16}$	0.075815	<$2.2\times 10^{-16}$	0.10402	<$2.2\times 10^{-16}$
Anderson-Darling statistic	38.602	$1.44\times 10^{-7}$	35.593	$1.44\times 10^{-7}$	36.201	$1.44\times 10^{-7}$	53.252	$1.44\times 10^{-7}$
Unit Leaving Fluid Temperature **(TFSU)**								
Kolmogorov-Smirnov statistic	0.0888	<$2.2\times 10^{-16}$	0.08	<$2.2\times 10^{-16}$	0.0821	<$2.2\times 10^{-16}$	0.1165	<$2.2\times 10^{-16}$
Anderson-Darling statistic	41.351	$1.44\times 10^{-7}$	33.063	$1.44\times 10^{-7}$	35.627	$1.44\times 10^{-7}$	92.955	$1.44\times 10^{-7}$

**Table 3:** Linear regression results.

	Lineal	Quadratic	Cubed	Exponential
**TAS**				
R2-ajus	−0.0002	−0.0004	−0.0007	−0.0002
MSE	0.1392	0.1392	0.1392	0.00035
F-statistic	0.000054	0.0034	0.0071	0.0017
*p*-value	0.9814	0.9966	0.9992	0.9663
**TAR**				
R2-ajus	0.1516	0.1515	0.2462	0.1521
MSE	0.041	0.041	0.03698	$5.8\times 10^{-5}$
F-statistic	742.8	371.6	453.1	745.5
*p*-value	<$2.2\times 10^{-16}$	<$2.2\times 10^{-16}$	<$2.2\times 10^{-16}$	<$2.2\times 10^{-16}$
**TFEU**				
R2-ajus	0.0168	0.01823	0.0541	0.01627
MSE	0.99	0.99	0.95	0.0078
F-statistic	71.93	39.54	80.28	69.68
*p*-value	<$2.2\times 10^{-16}$	<$2.2\times 10^{-16}$	<$2.2\times 10^{-16}$	<$2.2\times 10^{-16}$
**TFSU**				
R2-ajus	0.039	0.04154	0.11	0.04
MSE	0.75	0.748	0.6945	0.0031
F-statistic	171.5	90.97	172.9	171.8
*p*-value	<$2.2\times 10^{-16}$	<$2.2\times 10^{-16}$	<$2.2\times 10^{-16}$	<$2.2\times 10^{-16}$

**Table 4:** Estimated ARIMA models.

	TAR	TAS	TFEU	TFSU
ARIMA Model	(3,1,5)	(5,0,2) with non-zero mean	(5,1,2)	(5,1,1)
sigma${}^{2}$ estimated	0.01076	0.07068	0.3367	0.3367
log likelihood	3528.83	−388.69	−3522.97	−3522.97
AIC	−7039.65	795.38	4981.6	7059.94
BIC	−6982.65	852.39	5032.24	7104.05

**Table 5:** Dataset statistics.

Day	Src Bytes	Dst Bytes	Src Packets	Dst Packets	Prot_TCP	Prot_UDP	Prot_MQTT	Pack_Normal	Pack_Anomaly
Monday	699,527	348,336	9526	4905	13,936	495	9248	14,431	0
Tuesday	696,658	347,846	9491	4907	13,908	490	9184	14,398	0
Wednesday	702,714	350,844	9574	4950	14,032	492	9264	14,316	208
Thursday	703,431	351,018	9585	4953	14,048	490	9268	14,426	112
Friday	704,084	351,124	9592	4952	14,054	490	9292	14,160	384
Saturday	707,946	353,310	9645	4985	14,138	492	9339	14,246	384
Sunday	702,744	350,682	9571	4947	14,026	492	9277	14,518	0
**Total**	**4,917,104**	**2,453,160**	**66,984**	**34,599**	**98,142**	**3441**	**64,872**	**100,495**	**1088**

**Table 6:** Protocol composition.

Protocol		Monday	Tuesday	Wednesday	Thursday	Friday	Saturday	Sunday
TCP	MQTT	9248	9184	9264	9268	9292	9339	9277
		4688	4724	4768	4780	4762	4799	4749
UDP	NBNS	3	0	0	0	0	0	0
	NTP	492	490	492	490	490	492	492

**Table 7:** Number of flows.

Day	Total	Anomaly	Normal	TCP	UDP
Monday	9710	0	9710	9216	494
Tuesday	9666	0	9666	9176	490
Wednesday	9748	104	9644	9256	492
Thursday	9746	56	9690	9256	490
Friday	9762	192	9570	9272	490
Saturday	9828	192	9636	9336	492
Sunday	9388	0	9388	8896	492
**Total**	**67,848**	**544**	**67,304**	**64,408**	**3440**

**Table 8:** TCP flags analysis.

		Broker	
		S	SF
Client
	S	16	0
	SF	0	4
	SFR	7	0
	SFRR	0	85
